# Socio-Demographic Inequalities in Diagnostic Delays of Breast Cancer: A Multistage Time-to-Diagnosis Analysis

**DOI:** 10.3390/curroncol32120674

**Published:** 2025-12-01

**Authors:** Oana Maria Burciu, Tudor Gramada, Smaranda Gramada-Stefurac, Raluca-Alina Plesca, Cristina Macuc, Andreea-Lucia Viforeanu, Ioan Sas, Aida Iancu, Adrian-Grigore Merce, Ionut Marcel Cobec, Gabriel Mihail Dimofte

**Affiliations:** 1 Doctoral School, Faculty of Medicine, “Victor Babes” University of Medicine and Pharmacy Timisoara, 300041 Timisoara, Romania; 2 Doctoral School, Grigore T. Popa University of Medicine and Pharmacy Iasi, 700483 Iasi, Romania; 3 Department of Radiology, Regional Institute of Oncology, 700483 Iasi, Romania; 4 Department of Obstetrics and Gynecology, “Victor Babes” University of Medicine and Pharmacy Timisoara, 300041 Timisoara, Romania; 5 Department of Radiology, “Victor Babes” University of Medicine and Pharmacy Timisoara, 300041 Timisoara, Romania; 6 Department of Cardiology, Institute of Cardiovascular Diseases, 300310 Timisoara, Romania; 7 Department of Obstetrics and Gynecology, Faculty of Medicine, Medical Center-University of Freiburg, 79106 Freiburg, Germany; 8 Clinic of Obstetrics and Gynecology, Klinikum Freudenstadt, 72250 Freudenstadt, Germany; 9 Department of Surgery, Regional Institute of Oncology Iasi, Grigore T. Popa University of Medicine and Pharmacy Iasi, 700483 Iasi, Romania

**Keywords:** breast cancer screening, diagnostic delay, diagnostic intervals, timeliness, histopathological confirmation, rural–urban inequalities, socio-demographic factors, vulnerable populations, early detection

## Abstract

Background/Objectives: Introduction: Breast cancer remains a leading cause of cancer morbidity and mortality among women, and timely diagnosis is critical for improving outcomes. Organized screening programs strive to function efficiently, with minimal delays; however, evidence indicates that longer waiting times may be present at different stages of the diagnostic process. Few studies have evaluated how socio-demographic, reproductive, lifestyle, and clinical characteristics may influence diagnostic timeliness in a regional screening context. Materials and Methods: We retrospectively analyzed data from 240 women who underwent breast biopsy following abnormal screening assessment, out of 24,000 patients enrolled in a regional breast cancer screening program conducted in Northeastern and Southeastern Romania. Diagnostic timeliness was observed across three consecutive intervals of the screening pathway: mammography to biopsy (T1), biopsy to histopathological confirmation (T2), and cumulative presentation-to-diagnosis time (T3). Baseline population characteristics were described, subgroup comparisons performed, and multivariable regression models applied to identify independent predictors of diagnostic delay and to explore interaction effects at different stages of the screening process. Results: The interval between mammography and biopsy accounted for the most substantial waiting times (T1 median 24 days). The cumulative time to diagnosis (T3) reached a median of 32 days. Territorial inequalities were the strongest determinant of delay: rural patients experienced approximately five additional days before histopathological confirmation compared with urban patients (*p* = 0.003). Social vulnerability further contributed to prolonged T1 and T3 intervals, while lifestyle, reproductive, and anthropometric factors showed only minor or inconsistent associations. Interaction analyses revealed that delays linked to rural residence were most pronounced among younger women, an age group at higher risk for aggressive subtypes such as triple-negative breast cancer. Conclusions: In our findings, regional background and social vulnerability outweighed individual risk factors in shaping total diagnostic time. These results support the careful monitoring of interval-specific performance to strengthen equitable access to biopsy among vulnerable populations, where the effectiveness of early breast cancer detection is often challenged.

## Simple Summary

Breast cancer screening helps detect the disease early, when treatment is most effective. In this study, we analyzed data from nearly 24,000 women who participated in a regional screening program in Northeastern and Southeastern Romania, out of which 240 required biopsies for suspected lesions. By dividing the screening process into defined time intervals, we report that the primary delays occurred between mammography and biopsy, which contributed most to the overall diagnostic interval. In line with this trend, women living in rural areas and those identified as socially vulnerable tended to experience longer waits, especially younger women. These results emphasize the importance of further improving access to biopsy and follow-up, making breast cancer screening in the regional setting even more effective.

## 1. Introduction

Breast cancer is not only the most common malignancy among women worldwide but also one of the most heterogeneous in its presentation, prognosis, and response to treatment [1,2]. While the global burden of breast cancer is often expressed in terms of incidence and mortality, a less commonly discussed aspect is the persistent variation in stage at diagnosis across populations [3]. In countries with sustained declines in breast cancer mortality, early diagnosis is common, with at least 60% of invasive cancers detected at stage I or II. In contrast, numerous middle-income or low-income countries continue to report high proportions of advanced-stage breast cancer (stages III and IV) at time of diagnosis [4,5]. This variation reflects not only patient-level differences but, more importantly, systemic barriers to access to timely diagnosis and treatment in different socio-economic contexts. Moreover, studies report that even within countries with established screening programs, interval cancers (breast cancers diagnosed between scheduled screening rounds) account for 20–30% of cases, indicating limitations in both program adherence and diagnostic efficiency [6]. These patterns highlight that while incidence and mortality data are commonly used reference points in breast cancer statistics, they are insufficient to fully capture the extent of the challenges faced by healthcare systems worldwide in ensuring timely breast cancer detection.

Timely diagnosis is critical because every delay along the diagnostic pathway of breast cancer can contribute to disease progression, higher treatment complexity, and worse outcomes for patients. Recent systematic reviews and meta-analyses have addressed the problem of prolonged intervals within the breast cancer diagnostic route, which inevitably contribute to disease progression and advancement to a higher stage at the time of diagnosis [7,8]. In line with this, further delays in treatment initiation are associated with more intensive therapeutic measures, thereby worsening prognosis and increasing mortality risk, a finding that appears especially significant among younger women and in cases with more aggressive tumor subtypes [9]. Delays in the diagnostic pathway can arise from patient-level factors, such as symptom recognition or care-seeking, or from healthcare system constraints, including imaging, biopsy scheduling, and pathology reporting time. Understanding the possible bottlenecks of the diagnostic process—whether at the patient level or within the system—is particularly important in the context of organized screening programs, where timeliness is expected but not always ensured. Audits of European and North American screening initiatives have shown that diagnostic follow-up after abnormal mammography can vary widely, with median times ranging from about 10 days to 30 days, depending on program structure and resource availability [10,11].

The diagnostic trajectory of breast cancer is best conceptualized as a sequence of distinct but interconnected intervals, each representing a potential point of delay. Within organized screening programs, this pathway typically begins with the presentation to screening mammography, followed by the interval from diagnostic imaging to biopsy (T1), the period from biopsy to histopathological confirmation (T2), and finally the cumulative time to definitive diagnosis (T3) [7,11,12]. By breaking down the process in this manner, it becomes possible to assess not only the overall burden of diagnostic delay but also the specific stages most vulnerable to disruption. This stepwise approach of the breast cancer diagnostic route into clearly defined yet interrelated intervals is largely consistent with other recent literature assessing the timeliness of cancer diagnosis.

For example, reports from Canada and the United States have shown that the time from abnormal mammography to biopsy varies substantially across healthcare environments, with longer delays observed among women screened in mobile units and those belonging to racial and ethnic minority groups [10,13,14]. Similarly, multi-institutional analyses indicate that pathology processing times from biopsy to final report remain heterogeneous, reflecting differences in laboratory capacity and organizational efficiency between different centers [12,15]. Considering each interval separately is particularly relevant in the context of screening programs, where identifying the stages most vulnerable to delay can guide targeted interventions in order to improve both accessibility and program effectiveness [7,11].

In the existing literature, socio-demographic and territorial factors are associated with delays in breast cancer diagnosis, with rural residence, lower education level, and socio-economic disadvantage being the most frequently reported factors [11,13]. Reproductive and lifestyle factors such as number of births, contraceptive use, smoking status, and a higher body mass index (BMI) have also been implicated, although results are inconsistent and often context-specific [16]. Clinical characteristics, including age at diagnosis, tumor biology, and comorbidities, may further influence both the urgency of evaluation and the timeliness of follow-up. Most prior studies have examined these determinants in isolation or with limited consideration of underexplored variables that may influence both the risk factors and diagnostic outcomes. Recent evidence states that combined vulnerabilities—such as rural residence and low socio-economic status—can together influence diagnostic timeliness; however, few studies have formally examined these interaction effects [7,10,11].

Unlike national programs, regional initiatives reflect the realities of healthcare management and delivery within specific populations, where organizational structures, resource distribution, and demographic characteristics may differ considerably from national norms. Regional screening programs provide an opportunity to assess diagnostic timeliness in a practical context, where program resources are challenged by local system capacity and patient-level behaviors, offering valuable guidance for future growth. As a positive outcome, regional analyses can help uncover some of the healthcare shortcomings in lower-income areas and support targeted improvements such as building healthcare infrastructure, introducing mobile or community-based screening units and developing an integrated pathway, ultimately reducing diagnostic delays [7,11,13]

In Romania, organized breast cancer screening is not yet implemented at the national level but currently operates through regional initiatives coordinated by specialized oncology centers. These programs follow European guidelines for population-based screening and serve as practical models for evaluating screening logistics and diagnostic timeliness ahead of nationwide implementation.

In this context, we conducted a comprehensive evaluation of diagnostic timeliness within a regional breast cancer screening program. Our main goals were to (1) describe the baseline socio-demographic and clinical characteristics of screened women; (2) compare diagnostic intervals across relevant subgroups; (3) identify independent predictors of each interval using multivariable regression models; and (4) explore potential interaction effects between key predictors on the overall time from mammography to histopathological confirmation. By dividing the diagnostic trajectory into well-defined intervals, the present study aims to identify barriers and inequalities that may limit the effectiveness of screening programs in the regional setting and to quantify the extent to which specific factors, individually or combined, may influence diagnostic waiting time, especially among vulnerable populations.

## 2. Materials and Methods

In this study, we analyzed data from 240 women in need of breast biopsies out of 24,000 patients enrolled in a regional breast cancer screening program in Northeastern and Southeastern Romania coordinated by the Regional Institute of Oncology Iasi and conducted a comprehensive statistical analysis to investigate socio-demographic, territorial, reproductive, lifestyle, and clinical factors associated with diagnostic delays.

The inclusion criterion for age was established in alignment with international breast cancer screening guidelines, which designate women aged 50 to 69 years as the target population, reflecting the age group in which organized screening has demonstrated the greatest effectiveness in terms of early detection and mortality reduction.

The vulnerable population within the study cohort consisted of women from rural areas, particularly those residing in remote villages, single mothers, and individuals belonging to communities with markedly low socio-economic status.

Continuous variables were summarized using medians and interquartile ranges (IQRs), while categorical variables were summarized using absolute frequencies and percentages. For categorical variables derived from continuous measures, clinically and demographically meaningful cut-offs were applied. Age was grouped into three categories: <55, 55–64, and ≥65 years. From weight and height, we calculated body mass index (BMI)—weight (kg) divided by height squared (m_2_)—which was classified according to WHO categories (<25, 25–29.9, ≥30 kg/m^2^) and classified as underweight, normal, overweight, and obesity I–III. Menarche was categorized as ≤11 years (early), 12–14 years (normal), and ≥15 years (late). Age at first birth was grouped as <20, 20–24, 25–29, and ≥30 years, with a separate category for women with no births. Number of births was grouped as 0, 1, 2, and ≥3. Menopause status was categorized as early (<45 years), normal (45–54 years), and late (≥55 years). Smoking was dichotomized as “No” (non-smoker) versus “Yes” (smoker) (any level). The histopathological result variable was recoded as “Positive” versus “Negative.” For T3, additional categorical indicators were created to reflect timely diagnosis, defined as diagnosis within ≤14, ≤30, and ≤60 days.

The primary outcome was the time to diagnosis, operationalized through three sequential intervals: T1 (mammography to biopsy), T2 (biopsy to histopathology), and T3 (mammography to histopathology). T3 represents the cumulative diagnostic interval, including both T1 and T2, and was calculated as the total duration between the date of the initial screening mammography and the date of histopathological confirmation. The analysis aimed to (1) describe baseline characteristics of the study population, (2) compare diagnostic intervals across socio-demographic and clinical subgroups, (3) evaluate multivariable predictors of each diagnostic interval, and (4) explore interaction effects between key predictors on the overall time to diagnosis (T3).

Comparisons of continuous variables between groups were performed using the Mann–Whitney U test (two groups) or Kruskal–Wallis test (more than two groups), as non-normal distributions were confirmed using the Shapiro–Wilk test. Categorical variables were compared across groups using Pearson’s Chi-Squared test.

To evaluate predictors of diagnostic intervals, separate multivariable linear regression models were constructed for T1 through T3. Predictors were selected using the backward elimination method, guided by Akaike’s Information Criterion (AIC). Model performance was assessed using adjusted R^2^, and estimates with 95% confidence intervals (CI) and p-values were reported. 

Interaction effects between key socio-demographic variables were further examined using linear regression with interaction terms. Interaction plots were generated to visualize whether the effect of environment (urban vs. rural) on T3 was modified by vulnerability status or age group.

All analyses were conducted at a significance level of *p* < 0.05. Results were presented in tables and figures, with visualizations including boxplots and interaction plots to highlight inequalities in diagnostic delays across groups. Data analysis was performed using R (version 4.3.0) and RStudio (version 2023.06.0+421).

## 3. Results

The primary objective of the statistical analysis was to evaluate socio-demographic and territorial inequalities in diagnostic delays of breast cancer by examining three sequential time intervals: T1 (mammography to biopsy), T2 (biopsy to histopathology), and T3 (mammography to histopathological diagnosis). The dataset included 240 patients and incorporated socio-demographic variables (living environment, region, vulnerability status, age), anthropometric measures (weight, height), reproductive and hormonal factors (age at menarche, age at first birth, number of births, breastfeeding and its duration, age at menopause, menopausal hormone therapy), and lifestyle indicators (daily physical activity, alcohol consumption, and smoking status).

### 3.1. Baseline Characteristics of the Study Population

Across the biopsy cohort (*n* = 240), median diagnostic intervals were as follows (Table 1): the time from mammography to biopsy (T1) had a median of 24 days (IQR 15–30); the biopsy-to-histopathology interval (T2) was shorter at 8 days (IQR 2–12); and the cumulative time from mammography to histopathological result (T3) reached 32 days (IQR 25–40). These values indicate that the most substantial delay within the biopsy pathway accrues between mammography and biopsy.

The socio-demographic and clinical profile of the biopsy cohort is summarized in Table 2. Patients were more often urban (57.9%) than rural (42.1%), and 45.8% were identified as vulnerable. A large majority reported breastfeeding at some point (85.8%), while menopausal hormone therapy use was uncommon (4.2%). Lifestyle indicators showed daily physical activity in 21.2%, alcohol consumption in 18.3%, and smoking in 12.5%. As expected in a biopsy-selected sample, histopathological confirmation of malignancy was frequent (69.6%).

Anthropometric distribution revealed a high burden of excess weight: overweight (35.0%) and obesity (Obesity I, 29.2%; Obesity II, 15.0%; Obesity III, 3.8%) together accounted for 83.0% of the cohort; normal BMI comprised 16.7%, and underweight 0.4%. By age, 45.0% were 55–64 years, with 27.5% <55 and 27.5% ≥65. Reproductive markers showed menarche at 12–14 years in 66.8%, late menarche (≥15) in 25.0%, and early (<11) in 6.2%. Age at first birth clustered at 20–24 years (53.7%), with <20 in 17.5%, 25–29 in 11.7%, ≥30 in 9.6%, and no births in 7.5%. Parity was most commonly two births (40.8%), followed by ≥3 (26.7%), one (25.0%), and none (7.5%). Menopause was normal (45–54 years) in 67.9%, with not in menopause 13.3%, early (<45) 12.1%, and late (≥55) 6.7%. Collectively, this profile reflects a predominantly urban, high-BMI biopsy cohort with reproductive and menopausal characteristics consistent with regional patterns—context that informs subsequent analyses of socio-territorial inequalities in diagnostic timing.

### 3.2. Comparative Analysis of Diagnostic Delays Across Socio-Demographic and Clinical Factors

Clear inequalities appeared at T1 (mammography to biopsy) (Table 3). Patients from rural areas experienced longer delays compared to their urban counterparts (median 25 vs. 21 days, *p* = 0.007). Similarly, vulnerable patients (women from remote villages, single mothers, and individuals from communities with very low socio-economic status) faced extended waiting times relative to non-vulnerable patients (24 vs. 22 days, *p* = 0.040). Daily physical activity also showed a borderline association, with more active patients experiencing slightly longer delays (*p* = 0.050). No consistent associations were observed with anthropometric, reproductive, or lifestyle factors, suggesting that socio-territorial status exerts the strongest influence on this interval.

At T2 (biopsy to histopathology), overall delays were shorter, with median values between 7 and 8 days (Table 4). No major socio-demographic differences were detected. The only significant association was with pathology results: patients with positive histopathological diagnoses had longer delays compared to those with negative findings (9 vs. 5 days, *p* = 0.006). 

Finally, T3 (mammography to histopathological diagnosis) revealed cumulative socio-demographic inequalities (Table 5). Rural patients had significantly longer diagnostic intervals compared to urban patients (34 vs. 30 days, *p* = 0.003), and vulnerable patients faced delays relative to non-vulnerable patients (33 vs. 31.5 days, *p* = 0.020). These findings indicate that territorial and vulnerability-related factors not only influence intermediate steps but also translate into clinically meaningful cumulative delays. No significant associations were observed with lifestyle, reproductive, or anthropometric variables.

### 3.3. Multivariate Linear Regression Analysis of Diagnostic Delays (T1–T3)

The multivariate regression models provided further insight into the factors independently associated with diagnostic delays (Table 6, Table 7 and Table 8). 

For T1, daily physical activity was the only significant predictor; however, this association is unlikely to be clinically meaningful. Patients reporting regular physical activity experienced longer intervals between mammography and biopsy (β = 6.95 days, 95% CI 1.66–12.24, *p* = 0.010) (Table 6). The explanatory power of the model was limited (R^2^ = 0.023), indicating that additional unmeasured factors likely drive delays in this step.

In the T2 model (Table 7), age was an independent predictor. Women aged 55–64 years had shorter biopsy-to-histopathology intervals compared to those younger than 55 years (β = −2.28, 95% CI −4.41 to −0.15, *p* = 0.036). No other age effects reached statistical significance, and the model explained minimal variance (R^2^ = 0.010).

We hypothesized that longer reporting delays in the youngest patients in Group 1 could be explained by more frequent use of immunohistochemistry (46.7% vs. 39.9%) and 45.3% on their biopsies; to test this, we examined the association between age and immunohistochemistry (IHC) utilization. The data suggest a higher IHC frequency in the youngest cohort, but the difference is not statistically significant in the current sample—*p* = 0.300. Given the small effect size and limited precision, a larger cohort may provide sufficient power to determine whether this trend reflects a true association or sampling variability; at present, the finding remains trend-level rather than confirmatory.

Finally, for T3 (Table 8), environment was the key independent predictor. Patients from urban areas experienced shorter cumulative diagnostic delays compared to rural patients (β = −5.23, 95% CI −9.82 to −0.63, *p* = 0.026). Although the overall explanatory power remained modest (R^2^ = 0.017), this finding reinforces the role of territorial inequalities as a meaningful determinant of total diagnostic delay.

Taken together, these analyses reveal that while early steps of the diagnostic pathway (T1, T2) are largely uniform across patient groups, differences become more apparent in the cumulative trajectory to histopathological diagnosis (T3). The univariate analyses demonstrated that rural residence and vulnerability status consistently translated into longer delays, and regression models confirmed that environment remained an independent predictor of extended diagnostic intervals. Although some associations were detected for BMI, age, and lifestyle variables, these were modest in magnitude and lacked clinical relevance. Notably, the most substantial and persistent inequalities were territorial, with rural patients experiencing delays of approximately 5 additional days in obtaining a definitive diagnosis compared to their urban counterparts. These findings reveal the critical influence of socio-demographic context—particularly geographic location and vulnerability—on timely access to breast cancer diagnosis.

### 3.4. Interaction Effects of Environment with Socio-Demographic Factors on Diagnostic Delays

**Figure 1:**
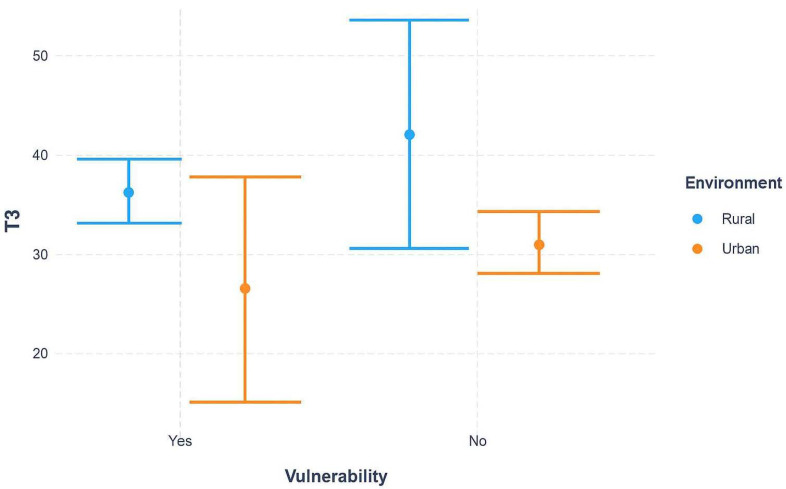
Interaction between environment and vulnerability status on cumulative diagnostic interval (T3).

Figure 1 illustrates the combined effect of environment (urban vs. rural) and vulnerability status on diagnostic delays (T3). Rural patients consistently experienced longer delays than their urban counterparts. Importantly, the gap between rural and urban settings was more pronounced among non-vulnerable patients, whereas vulnerable patients showed broadly similar delays across environments, with wide confidence intervals suggesting variability and uncertainty. This pattern highlights that geographic inequities persist independently of vulnerability, but vulnerability may further complicate or mask the effect of environment in some subgroups.

Median time from mammography to histopathological confirmation (T3) was stratified by urban versus rural residence and vulnerability status. Rural patients consistently experienced longer delays compared with their urban counterparts. The difference was more pronounced among non-vulnerable women, whereas among vulnerable women, delays were substantial across both environments. Error bars represent interquartile ranges (IQR).

**Figure 2:**
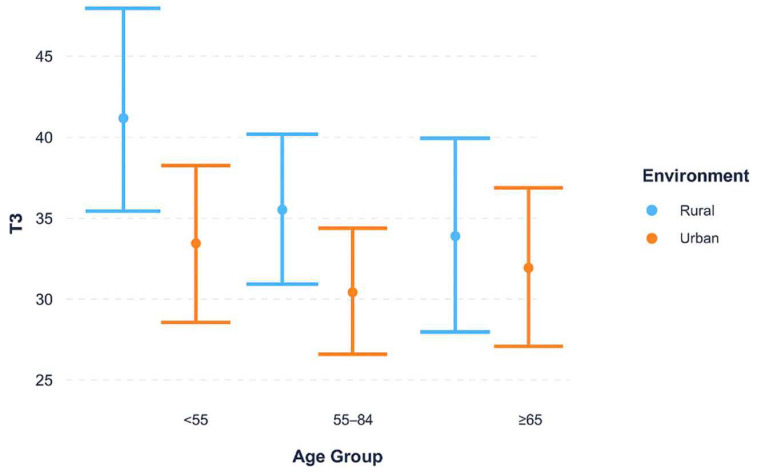
Interaction between environment and age group on cumulative diagnostic interval (T3).

Figure 2 examines whether the urban–rural diagnostic delay differs across age groups. Across all age strata, rural patients experienced longer delays compared to urban patients. The magnitude of this difference was most evident among younger patients (<55 years), with the gap narrowing in older age groups. These findings suggest that geographic differences in diagnostic timeliness are notably relevant for younger women.

Median time from biopsy to histopathological confirmation (T3) was stratified by urban versus rural residence across age categories (<55, 55–64, ≥65 years). Rural patients demonstrated longer delays in all stratifications, with the largest urban–rural gap observed among women under 55 years. This pattern indicates that differences in diagnostic timeliness may be more evident in populations living in remote rural areas, where access to diagnostic services is more time-consuming, whereas delays in younger women may reflect differences in care-seeking or diagnostic complexity, though this cannot be confirmed from our data (IQR).

The interaction analyses reinforce that diagnostic delays in breast cancer are shaped not only by individual socio-demographic factors but also by their interplay with the broader environment. Rural residence systematically amplified delays across both vulnerable and non-vulnerable populations, with the effect being most pronounced among younger women. These findings emphasize the dual burden of geographic and demographic inequalities, suggesting that interventions targeting early diagnostic pathways must address both structural barriers in rural healthcare provision and the specific needs of socially disadvantaged groups.

## 4. Discussion

### 4.1. Principal Findings

In order to efficiently evaluate the overall time to diagnosis and the potential socio-demographic factors influencing it, the process was divided into three sequential time intervals: T1 (mammography to biopsy), T2 (biopsy to histopathology), and T3 (mammography to histopathology). The first interval (T1) represented the principal challenge in the screening process, accounting for the majority of the total waiting time, approaching three to four weeks (25 days). The median duration of the second interval (T2) was shorter, 8 days, with slightly longer delays in patients with positive histopathological diagnoses vs. patients with negative findings (9 vs. 5 days), likely reflecting increased complexity of processing malignant specimens. Taken together, these intervals resulted in an overall median waiting time (T3) of 32 days.

The characteristics of the study population add important context for understanding the diagnostic timelines observed. The statistical analysis showed that the population was substantially rural (42%), socially vulnerable (46%), and predominantly overweight or obese (83%), reflecting the very groups that organized screening programs are designed to reach. These characteristics mirror the demographic profile of the region and may help explain variation in diagnostic timeliness.

These baseline population characteristics were mirrored in our findings, where territorial differences emerged as the most consistent and clinically consistent determinant of delay. Rural residence was associated with approximately five additional days before histopathological confirmation, a gap that persisted even after multivariable adjustment. Vulnerability status further amplified these delays, indicating that inequalities are not only geographic but also socio-structural. By comparison, reproductive characteristics, lifestyle indicators, and anthropometric measures—although occasionally reaching statistical significance—showed minimal effect sizes and are unlikely to carry clinical relevance.

An additional factor that may have contributed to diagnostic delays relates to the organizational workflow of the screening program. Most mammograms were performed within the central monitoring facility, where both double reading and arbitration were conducted. While this approach ensured consistent quality control, it may have also generated additional waiting times, particularly for women residing in the southern regions, where the mobile unit had to return to base before image review could be completed. These logistical aspects should be considered as a possible contributor to the observed regional differences, although they were not directly assessed in this study.

Finally, the interaction analyses reflect that inequalities are not merely additive but intersectional. Rural delays were evident in both vulnerable and non-vulnerable subgroups, highlighting the complex nature of inequities. However, delays attributable to rural residence were most pronounced among younger women, a group in which early diagnostic resolution is especially critical. Beyond structural barriers, prior literature suggests that the lower perceived risk of cancer in this age group and lower referral urgency could potentially lead to extended diagnostic intervals.

### 4.2. Comparison with Existing Literature

Our findings are in line with several recent international studies demonstrating that geographic and socio-demographic inequalities continue to influence timeliness of breast cancer diagnosis, providing context for our results. Vedsted and colleagues reported in a comparative study, which targeted symptomatic women with breast cancer, that the median diagnostic interval, from presentation to confirmed diagnosis, ranged from 8 days in Denmark to 29 days in Wales. While our cumulative diagnostic trajectory of 32 days is not directly comparable in design, it falls close to the upper end of these ranges [17]. In Switzerland, the Swiss Donna mammography screening program reported diagnostic advantages reflected by earlier-stage detection and a significantly improved survival among screened women. This aligns with findings from the ICBP study by Vedsted et al., which showed that jurisdictions with more efficient diagnostic pathways—Sweden included—achieve faster diagnostic intervals and better breast cancer outcomes overall. Together, these studies emphasize how organized screening and timely diagnosis contribute to improved survival [17,18].

Evidence from Asia also offers perspective. In a report from Malaysia, the median time from first presentation to confirmed breast cancer diagnosis was 26 days, with substantial variability across patients. This result is in line with our cumulative diagnostic trajectory of 32 days and emphasizes that system-level factors significantly affect timeliness [19,20]. Similarly, in a study from US-accredited breast centers, it was reported that the combined time from screening mammogram to biopsy was approximately 19–21 days, largely similar to the 25-day mammography-to-biopsy interval observed in our cohort. Notably, whereas the longest waiting time in the US occurred after biopsy during the transition to treatment, our findings identified the mammography-to-biopsy step as the most substantial source of delay within the diagnostic pathway [21]. 

Although Romania is also classified as a middle-income country, the median diagnostic interval of 32 days observed in our study was considerably shorter than the months-long delays frequently reported in low- and middle-income countries (LMICs), as reported by other scientific works. This contrast suggests that, although Romania still faces challenges in diagnostic timeliness, even in resource-constrained settings, the presence of an organized screening program offers encouraging evidence of what can be achieved in a middle-income context [22]. Table 9 below summarizes the main findings of the studies cited in this section, providing an overview of reported diagnostic intervals across different settings.

Complementing this perspective, one study from Morocco identified multiple determinants of delayed diagnosis in breast cancer patients, showing that socio-demographic and systemic barriers to timely confirmation are present in both screening and symptomatic populations [23]. Agodirin et al. further showed that in Nigeria, both structural challenges within the health system and patient-related factors, such as limited awareness and reliance on alternative care, contributed to prolonged diagnostic delays and presentation at more advanced stages of disease [24,25]. Similarly, an American population-based study reported that women residing in rural areas were more likely to be diagnosed at later stages of breast cancer in comparison to urban-located women, illustrating that geographic inequalities in stage at presentation can be present even in high-income health systems [26]. In line with these findings, a review article, which summarizes evidence of geographic differences in breast cancer, emphasized that factors such as rural residence and living in low-income communities contribute to delayed detection, later stage at diagnosis, and poorer outcomes [27].

Our observation regarding extended diagnostic intervals in rural populations aligns with recent studies from the United States (USA). One investigation reported that rural residence and living more than 40 miles from a diagnostic center significantly increased delays in biopsy after abnormal mammography [28]. In another U.S. publication that analyzed the cancer screening process, the conclusions reached were that rural women had 14–27% lower odds of meeting breast cancer screening recommendations compared with urban women, reflecting persistent barriers in early detection and prevention [29]. Delays at one stage of the care route can cascade into subsequent stages. For example, a U.S. study focusing on treatment intervals rather than diagnostic intervals found that breast cancer patients treated in rural hospitals experienced longer times to treatment initiation than those treated in urban hospitals, supporting our findings that geographic inequalities consistently influence timeliness across the cancer care route [30]. 

Even a study centered on Australian cancer survivors highlights that rural patients often reported traveling long distances for care and described more fragmented care routes and less direct diagnostic routes, factors likely to reduce adherence and contribute to diagnostic delays [31].

In addition to geographic factors, individual risk factors such as obesity may also further influence diagnostic timeliness. Retrospective cohort studies have shown that obesity is associated with reduced adherence to mammographic screening recommendations and variations in long-term screening patterns. These differences may contribute to delays later in the path to diagnosis and potentially affect clinical outcomes [32]. More recent analyses suggest that obesity complicates case management, influencing both timeliness and treatment planning [33].

Age also emerged as a relevant factor in our study. Rural residence had the strongest impact on diagnostic delays among women under 55, with differences becoming less pronounced in older age groups. This resonates with prior reports from symptomatic cohorts, where younger age was linked to delayed diagnostic resolution due to misattribution of symptoms or lower urgency of referral [34]. Such diagnostic challenges are especially concerning given that younger women are also more likely to present with aggressive tumor subtypes, which are associated with poorer prognoses. One retrospective study divided patients into groups of <40 and >40 years and found no statistically significant differences in overall survival or disease-free survival during the 5-year follow-up [35,36].

An intersectional perspective highlights that focusing on a single factor, such as gender or residence, overlooks how disadvantages often overlap in shaping access to care in a timely manner [37]. Scholars have also argued that broad categories like ‘women and minorities’ can hide important differences within groups, and that inequalities should instead be examined through the combined effects of multiple social positions [38]. Evidence supports this approach, showing that breast cancer screening experiences differ when race, ethnicity, and gender are considered together [39]. In this context, our findings that rural residence interacts with both age and vulnerability status to extend diagnostic delays suggest that interacting social and demographic factors play a critical role in shaping timeliness along the diagnostic pathway. 

Both patient-level and system-level determinants have been shown to influence timeliness and survival, and even modest reductions in delay may result in meaningful clinical benefits. Seminal analyses demonstrated that diagnostic delays surpassing three months were associated with worse survival [40]. More recent European and Australian reports confirmed that inequalities persisted across the breast cancer care pathway, despite structured screening programs, reflecting the meaningful impact of geographic and social disadvantage within healthcare systems [41,42]. Another recent European-based study indicated that design variability and regional implementation of breast cancer screening programs across Europe notably influenced screening performance, as supported by differences in key indicators and associated mortality outcomes [43]. 

### 4.3. Interpretation and Implications

Establishing the main contributor to overall diagnostic time provides an opportunity for more precise monitoring, which would not be possible if only cumulative timeliness is assessed. By decomposing the diagnostic process into separate intervals (T1–T3), we were able to identify which stages of the trajectory in our study were most sensitive to delay. These findings are also significant in the context of future screening settings.

The diagnostic intervals analyzed in our study (T1–T3) align with internationally recognized performance indicators for evaluating breast cancer screening programs. Timeliness between key diagnostic steps has been proposed as a core measure of program quality, as it reflects both organizational efficiency and the potential for early detection [44]. 

Although the adjusted R^2^ values of the multivariable models were modest, this is expected in population-based screening research, where diagnostic delay is influenced by numerous unmeasured system-level and behavioral factors. 

At the same time, evidence from alternative diagnostic approaches shows potential ways for improvement. The implementation of rapid diagnostic centers, for instance, has been shown to sustain timely breast cancer diagnosis even during pandemic times [45], suggesting that similar approaches may help reduce the geographic inequalities and are a promising strategy.

### 4.4. Strengths and Limitations

By simultaneously assessing socio-demographic, reproductive, lifestyle, and clinical factors, this study offers a detailed picture of the regional breast cancer screening practice in Romania. A distinctive feature of this work is the sequential, interval-based approach (T1–T3), which allowed delays to be identified with more precision at each stage of the screening process. In addition, the use of interaction analyses generated novel insights into how geographical and social contexts intersect to influence diagnostic time. To our knowledge, this is the first study of its kind in the region, making it uniquely positioned to guide strategies for strengthening timely and equal access to diagnosis.

This study has several limitations. As the data were retrospectively analyzed, causal interpretation is limited, and the explanatory power of regression models was modest. It is likely that additional system-level factors—such as diagnostic capacity, staffing, or referral practices—also played a role in shaping delays but could not be fully captured in this dataset. 

The analysis focused on variables previously reported in the literature as relevant to diagnostic delay. Other contextual factors, such as education level, socio-economic status, or biopsy technique, were beyond the scope of the present analysis; although their exclusion represents a limitation, it is unlikely to have substantially affected the observed trends.

Lastly, because the analytic cohort consisted only of women undergoing biopsy, the prevalence of malignancy was higher than in the overall screened population. This approach, while potentially introducing a degree of selection bias toward patients with higher suspicion of malignancy, accurately reflects the functioning of regional screening programs. Therefore, it supports the applicability of the findings in clinical practice.

## 5. Conclusions

In summary, this study shows that rural residence and social vulnerability are the main determinants associated with diagnostic delay within an organized regional breast cancer screening program, with the period of time between the mammography and biopsy responsible for the greatest overall diagnostic delay. Interaction analyses further showed that rural–urban differences in overall diagnostic delay were most pronounced among younger women, identifying a subgroup at particular risk of disadvantage. Assessing diagnostic timeliness through sequential interval-specific analysis is essential and highlights the need for tailored strategies for vulnerable populations and improved access to biopsy services. By building on the strengths of existing screening processes while addressing systemic barriers such as geographic access, healthcare infrastructure, and socio-economic factors, programs can both preserve the mortality benefit of early detection and ensure fair access to care in clinical practice.

## Figures and Tables

**Table 1:** Distribution of diagnostic delays across the four time intervals (T1–T3).

Variable	Median (Q25–Q75)
T1	24 (15–30)
T2	8 (2–12)
T3	32 (25–40)

Abbreviations: T1—Time from mammography to biopsy (days); T2—Time from biopsy to histopathological result (days); T3—Time from mammography to histopathological result (days).

**Table 2:** Socio-demographic, anthropometric, reproductive, lifestyle, and clinical characteristics of the study population (*n* = 240).

Variable	Group	N (%)
Environment	Rural	101 (42.1%)
	Urban	139 (57.9%)
Vulnerability	Yes	110 (45.8%)
Breastfeeding	Yes	206 (85.8%)
HTM	Yes	10 (4.2%)
DPA	Yes	51 (21.2%)
Alcohol	Yes	44 (18.3%)
Smoker	Yes	30 (12.5%)
Pathology	Positive	167 (69.6%)
BMI	Underweight	1 (0.4%)
	Normal	40 (16.7%)
	Overweight	84 (35.0%)
	Obesity I	70 (29.2%)
	Obesity II	36 (15.0%)
	Obesity III	9 (3.8%)
Age	< 55 years	66 (27.5%)
	55–64 years	108 (45.0%)
	≥65	66 (27.5%)
Menarche	<11 years	15 (6.2%)
	12–14 years	165 (66.8%)
	≥15	60 (25.0%)
Age 1st birth	No births	18 (7.5%)
	<20	42 (17.5%)
	20–24	129 (53.7%)
	25–29	28 (11.7%)
	≥30	23 (9.6%)
NO Births	0	18 (7.5%)
	1	60 (25.0%)
	2	98 (40.8%)
	3 or more	64 (26.7%)
Menopause	No	32 (13.3%)
	Early	29 (12.1%)
	Normal	163 (67.9%)
	Late	16 (6.7%)

Abbreviations: HTM—Hormone therapy for menopause; DPA—Daily physical activity; BMI—Body Mass Index; Pathology (Positive)—Histopathological confirmation of malignancy; Underweight—BMI < 18.5 kg/m^2^; Normal—BMI 18.5–24.9 kg/m^2^; Overweight—BMI 25.0–29.9 kg/m^2^; Obesity I—BMI 30.0–34.9 kg/m^2^; Obesity II—BMI 35.0–39.9 kg/m^2^; Obesity III—BMI ≥ 40.0 kg/m^2^; Menarche—Age at first menstruation; Age 1st birth—Maternal age at first childbirth; No births—Nulliparous women; Menopause (No)—Women not in menopause at study entry; Early menopause—Menopause before 45 years; Normal menopause—Menopause between 45 and 54 years; Late menopause—Menopause at or after 55 years.

**Table 3:** Distribution of T1 (mammography to biopsy) across socio-demographic and clinical factors.

Variable	Group	T1 Median (IQR)	*p*-Value
Environment	Rural	25.00 (19.25–33.00)	0.007
	Urban	21.00 (14.00–27.50)	
Vulnerability	Yes	24.00 (19.00–33.00)	0.040
	No	22.00 (15.00–28.00)	
Breastfeeding	Yes	24.00 (15.50–29.50)	0.590
	No	21.00 (15.00–26.25)	
HTM	Yes	20.50 (15.50–27.50)	0.620
	No	24.00 (15.00–30.00)	
DPA	Yes	25.00 (19.50–33.00)	0.050
	No	22.00 (15.00–29.00)	
Alcohol	Yes	21.00 (19.00–28.00)	0.980
	No	24.00 (15.00–30.00)	
Smoker	Yes	23.50 (18.25–27.75)	0.880
	No	24.00 (15.00–30.50)	
Pathology	Positive	22.00 (15.00–29.00)	0.060
	Negative	25.00 (20.00–32.00)	
BMI	Underweight	16.00 (16.00–16.00)	0.650
	Normal	24.00 (16.50–29.25)	
	Overweight	22.00 (15.00–30.50)	
	Obesity I	25.00 (15.00–28.50)	
	Obesity II	25.00 (18.00–32.00)	
	Obesity III	15.00 (11.00–27.00)	
Age	<55 years	22.00 (16.50–32.00)	0.830
	55–64 years	23.00 (15.00–29.00)	
	≥65	25.00 (15.00–30.50)	
Menarche	<11 years	26.00 (16.00–31.00)	0.800
	12–14 years	24.00 (16.00–30.00)	
	≥15	21.00 (15.00–27.25)	
Age 1st birth	No births	25.00 (14.25–32.75)	0.140
	<20	28.00 (20.25–32.00)	
	20–24	22.00 (15.00–29.00)	
	25–29	24.00 (15.75–32.00)	
	≥30	21.00 (10.50–26.00)	
NO Births	0	25.00 (14.25–32.75)	0.790
	1	22.50 (14.00–28.00)	
	2	23.50 (16.00–28.00)	
	3 or more	24.00 (15.00–32.00)	
Menopause	No	20.50 (13.75–26.50)	0.730
	Early	26.00 (17.00–29.00)	
	Normal	24.00 (15.00–32.00)	
	Late	24.50 (19.00–34.25)	

Abbreviations: T1—Time from mammography to biopsy(days); HTM—Hormone therapy for menopause; DPA—Daily physical activity; BMI—Body Mass Index; Underweight—BMI < 18.5 kg/m^2^; Normal—BMI 18.5–24.9 kg/m^2^; Overweight—BMI 25.0–29.9 kg/m^2^; Obesity I—BMI 30.0–34.9 kg/m^2^; Obesity II—BMI 35.0–39.9 kg/m^2^; Obesity III—BMI ≥ 40.0 kg/m^2^; Menarche—Age at first menstruation; Age 1st birth—Maternal age at first childbirth; No births—Nulliparous women; Menopause (No)—Women not in menopause at study entry; Early menopause—Menopause before 45 years; Normal menopause—Menopause between 45 and 54 years; Late menopause—Menopause at or after 55 years.

**Table 4:** Distribution of T2 (biopsy to histopathology) across socio-demographic and clinical factors.

Variable	Group	T2 Median (IQR)	*p*-Value
Environment	Rural	7.00 (2.00–13.00)	0.790
	Urban	8.00 (0.50–12.00)	
Vulnerability	Yes	7.00 (2.00–13.00)	0.800
	No	8.00 (0.25–12.00)	
Breastfeeding	Yes	7.00 (1.00–12.00)	0.390
	No	10.50 (3.00–14.00)	
HTM	Yes	6.00 (0.75–11.25)	0.570
	No	7.50 (2.00–13.00)	
DPA	Yes	6.00 (2.00–11.50)	0.480
	No	8.00 (0.75–13.00)	
Alcohol	Yes	8.00 (0.00–11.25)	0.770
	No	8.00 (2.00–13.00)	
Smoker	Yes	8.50 (2.50–15.00)	0.810
	No	7.00 (2.00–12.00)	
Pathology	Positive	9.00 (2.00–13.00)	0.006
	Negative	5.00 (0.00–9.00)	
BMI	Underweight	7.00 (7.00–7.00)	0.990
	Normal	8.00 (0.00–12.25)	
	Overweight	7.50 (2.00–12.25)	
	Obesity I	7.00 (0.25–13.00)	
	Obesity II	7.00 (2.50–10.00)	
	Obesity III	10.00 (4.00–13.00)	
Age	<55 years	8.50 (3.25–14.00)	0.210
	55–64 years	7.00 (0.00–11.50)	
	≥65	7.00 (0.00–13.75)	
Menarche	<11 years	7.00 (0.50–11.00)	0.830
	12–14 years	7.50 (2.00–13.00)	
	≥15	8.00 (0.00–12.00)	
Age 1st birth	No births	8.50 (2.25–12.75)	0.690
	<20	7.00 (0.00–11.00)	
	20–24	7.00 (1.75–12.00)	
	25–29	9.00 (3.00–13.25)	
	≥30	7.00 (3.00–13.50)	
NO Births	0	8.50 (2.25–12.75)	0.830
	1	8.50 (0.00–14.00)	
	2	7.00 (0.00–12.00)	
	3 or more	7.00 (2.00–11.25)	
Menopause	No	8.50 (2.00–13.25)	0.720
	Early	8.00 (4.00–12.00)	
	Normal	7.00 (0.00–12.00)	
	Late	8.50 (0.00–14.25)	

Abbreviations: T2—Time from biopsy to histopathology (days); HTM—Hormone therapy for menopause; DPA—Daily physical activity; BMI—Body Mass Index; Underweight—BMI < 18.5 kg/m^2^; Normal—BMI 18.5–24.9 kg/m^2^; Overweight—BMI 25.0–29.9 kg/m^2^; Obesity I—BMI 30.0–34.9 kg/m^2^; Obesity II—BMI 35.0–39.9 kg/m^2^; Obesity III—BMI ≥ 40.0 kg/m^2^; Menarche—Age at first menstruation; Age 1st birth—Maternal age at first childbirth; No births—Nulliparous women; Menopause (No)—Women not in menopause at study entry; Early menopause—Menopause before 45 years; Normal menopause—Menopause between 45 and 54 years; Late menopause—Menopause at or after 55 years.

**Table 5:** Distribution of T3 (mammography to histopathological diagnosis) across socio-demographic and clinical factors.

Variable	Group	T3 Median (IQR)	*p*-Value
Environment	Rural	34.00 (27.00–43.00)	0.003
	Urban	30.00 (21.00–36.00)	
Vulnerability	Yes	33.00 (27.00–42.00)	0.020
	No	31.50 (21.00–36.75)	
Breastfeeding	Yes	32.00 (25.00–39.00)	0.660
	No	35.50 (25.25–40.00)	
HTM	Yes	32.50 (20.00–34.50)	0.400
	No	32.00 (25.00–40.25)	
DPA	Yes	34.00 (26.00–40.00)	0.170
	No	31.50 (23.00–39.25)	
Alcohol	Yes	33.00 (24.50–39.25)	0.810
	No	32.00 (25.00–40.50)	
Smoker	Yes	32.50 (28.25–35.00)	0.990
	No	32.00 (23.00–41.00)	
Pathology	Positive	32.00 (23.00–40.00)	0.610
	Negative	33.00 (25.00–39.00)	
BMI	Underweight	23.00 (23.00–23.00)	0.720
	Normal	32.50 (24.50–42.00)	
	Overweight	32.00 (25.00–40.50)	
	Obesity I	31.00 (25.00–39.75)	
	Obesity II	33.00 (26.50–39.00)	
	Obesity III	29.00 (13.00–36.00)	
Age	<55 years	34.00 (26.25–41.00)	0.250
	55–64 years	31.00 (23.00–37.50)	
	≥65	33.00 (25.00–39.00)	
Menarche	<11 years	33.00 (24.00–37.50)	0.960
	12–14 years	32.50 (25.00–40.00)	
	≥15	31.00 (25.00–40.25)	
Age 1st birth	No births	34.50 (22.00–40.00)	0.430
	< 20	32.00 (28.00–38.00)	
	20–24	32.00 (25.00–39.25)	
	25–29	34.50 (26.00–45.50)	
	≥30	26.00 (18.00–38.00)	
NO Births	0	34.50 (22.00–40.00)	0.930
	1	29.50 (24.50–39.25)	
	2	33.00 (25.00–39.00)	
	3 or more	33.00 (25.75–42.25)	
Menopause	No	31.00 (21.00–41.25)	0.490
	Early	33.00 (21.00–41.00)	
	Normal	32.00 (25.00–39.00)	
	Late	33.00 (25.00–51.00)	

Abbreviations: T3—Time from mammography to histopathology (days); HTM—Hormone therapy for menopause; DPA—Daily physical activity; BMI—Body Mass Index; Underweight—BMI < 18.5 kg/m^2^; Normal—BMI 18.5–24.9 kg/m^2^; Overweight—BMI 25.0–29.9 kg/m^2^; Obesity I—BMI 30.0–34.9 kg/m^2^; Obesity II—BMI 35.0–39.9 kg/m^2^; Obesity III—BMI ≥ 40.0 kg/m^2^; Menarche—Age at first menstruation; Age 1st birth—Maternal age at first childbirth; No births—Nulliparous women; Menopause (No)—Women not in menopause at study entry; Early menopause—Menopause before 45 years; Normal menopause—Menopause between 45 and 54 years; Late menopause—Menopause at or after 55 years.

**Table 6:** Linear regression analysis of predictors for T1 (mammography to biopsy).

Predictors	Estimates	CI	P
DPA [Yes]	6.95	1.66−12.24	0.010
N = 240			
R^2^ = 0.023			

Abbreviations: T1—Time from mammography to biopsy (days); DPA—Daily physical activity.

**Table 7:** Linear regression analysis of predictors for T2 (biopsy to histopathology).

Predictors	Estimates	CI	P
Age Group (55–64)	−2.28	−4.41–−0.15	0.036
Age Group (≥65]	−1.59	−3.96–−0.78	0.188
N = 240			
R^2^ = 0.010			

Abbreviations: T2—Time from biopsy to histopathological result (days).

**Table 8:** Linear regression analysis of predictors for T3 (mammography to histopathological diagnosis).

Predictors	Estimates	CI	P
Environment [Urban]	−5.23	−9.82–−0.63	0.026
N = 240			
R^2^ = 0.017			

Abbreviations: T3—Time from mammography to histopathological result (days).

**Table 9:** Comparison of diagnostic intervals in breast cancer across selected studies.

Study/Country	Study Type/Population	Diagnostic Interval (s) Assessed	Median Waiting Time (Days)	Key Observations/Remarks
Vedsted P et al., 2022 [17]	International comparative cohort study in 10 jurisdictions (symptomatic women diagnosed with breast cancer)	First presentation to confirmed diagnosis	8–29 median; Denmark → Wales)	Based on ICBP Module 4; diagnostic interval defined as time from first presentation to confirmed diagnosis.
Mujar et al., 2017 (Malaysia) [19]	Hospital-based cohort (public hospitals)	First presentation to confirmed diagnosis	26	Demonstrated patient- and system-level variability in diagnostic timeliness; highlights role of healthcare accessibility and awareness.
Lawson et. al., 2022 (USA) [10]	Multi-institutional cohort (screening programs)	Abnormal screening mammogram to biopsy	~14–16	Median time to biopsy varied by race and ethnicity; variation reflects program-level and equity factors.
Ruco et.al., 2023 (Canada) [11]	Multicenter retrospective cohort (5 provinces)	Screening result requiring further assessment to confirm diagnosis	~26–30	Significant interprovincial variation; diagnostic timeliness influenced by healthcare system organization.
Current study (Romania)	Regional screening program (Northeast and Southeast Romania)	Mammography to histopathological diagnosis (T3 cumulative)	32	Identifies mammography-to-biopsy as key delay source; aligns with upper range of international findings.

Abbreviations: ICBP = International Cancer Benchmarking Partnership.

## Data Availability

Further information concerning the present article is available from the corresponding author upon reasonable request.
